# Folate Carrier Deficiency Drives Differential Methylation and Enhanced Cellular Potency in the Neural Plate Border

**DOI:** 10.3389/fcell.2022.834625

**Published:** 2022-07-13

**Authors:** Nagif Alata Jimenez, Pablo H. Strobl-Mazzulla

**Affiliations:** Laboratory of Developmental Biology, Instituto Tecnológico de Chascomús (CONICET-UNSAM), Escuela de Bio y Nanotecnologías (UNSAM), Chascomús, Argentina

**Keywords:** folate, neural plate, neural plate border, DNA methylation, NOTCH1

## Abstract

The neural plate border (NPB) of vertebrate embryos segregates from the neural and epidermal regions, and it is comprised of an intermingled group of multipotent progenitor cells. Folate is the precursor of S-adenosylmethionine, the main methyl donor for DNA methylation, and it is critical for embryonic development, including the specification of progenitors which reside in the NPB. Despite the fact that several intersecting signals involved in the specification and territorial restriction of NPB cells are known, the role of epigenetics, particularly DNA methylation, has been a matter of debate. Here, we examined the temporal and spatial distribution of the methyl source and analyzed the abundance of 5mC/5 hmC and their epigenetic writers throughout the segregation of the neural and NPB territories. Reduced representation bisulfite sequencing (RRBS) on Reduced Folate Carrier 1 (RFC1)-deficient embryos leads to the identification of differentially methylated regions (DMRs). In the RFC1-deficient embryos, we identified several DMRs in the *Notch1* locus, and the spatiotemporal expression of *Notch1* and its downstream target gene *Bmp4* were expanded in the NPB. Cell fate analysis on folate deficient embryos revealed a significant increase in the number of cells coexpressing both neural (SOX2) and NPB (PAX7) markers, which may represent an enhancing effect in the cellular potential of those progenitors. Taken together, our findings propose a model where the RFC1 deficiency drives methylation changes in specific genomic regions that are correlated with a dysregulation of pathways involved in early development such as Notch1 and BMP4 signaling. These changes affect the potency of the progenitors residing in the juncture of the neural plate and NPB territories, thus driving them to a primed state.

## Introduction

During gastrulation in amniote embryos, the bilaminar embryo forms three germ layers, with noningressing superficial epiblast cells forming the ectoderm. Then, the ectoderm undergoes a refined sequential territorial segregation to generate precursors for three major regions. The definitive neural plate (NP), which will form the central nervous system, is specified in the anteromedial territory (Fernández-Garre et al., 2002; Rex et al., 1997; Sanchez-Arrones et al., 2012; Streit et al., 1997; Uchikawa et al., 2003). Lateral to the NP, the neural plate border (NPB), which will become the neural crest and sensory placodes, is defined (Streit, 2007; Patthey and Gunhaga, 2011; Groves and LaBonne, 2014; Saint-Jeannet and Moody, 2014; Moody and LaMantia, 2015). The nonneural ectoderm (NNE) is found lateral to the NPB and will become the *epidermis*. The success of vertebrate evolution is highly dependent on the acquisition of the two cell fates residing in the NPB: the neural crest and placode cells. Both of these cells contribute to many of the synapomorphic characteristics of vertebrates, including a well-defined head with sensory organs and peripheral ganglia. Particularly, the neural crest cells differentiate into sensory and autonomic ganglia, pigment cells, and elements of the craniofacial skeleton, whereas ectodermal placodes give rise to the ear, nose, lens, and sensory ganglia of the head.

NBP induction involves an intersection between Bone Morphogenetic Protein (BMP), Fibroblast Growth Factor (FGF), and Wingless/Integrated (WNT) signaling. Together with those signaling molecules that are secreted from the future *epidermis*, the neural plate and mesoderm activate the expression of downstream transcription factors that are broadly conserved in vertebrates (Basch et al., 2000; Murdoch et al., 2010; Murdoch et al., 2012; Milet et al., 2013). Although there is an apparent heterogeneity in the progenitors residing in the NPB, these cells retain much of the pluripotent factors throughout gastrula and early neurulation stages, endowing them with the stem cell-like properties (Buitrago-Delgado et al., 2015; Hintze et al., 2017; Lignell et al., 2017; Roellig et al., 2017; Trevers et al., 2017; Buitrago-Delgado et al., 2018; Williams et al., 2019). This potential is evidenced by the simultaneous expression of neural, neural crest, and epithelial markers (SOX2/PAX7/SIX1) at the NPB domain (Roellig et al., 2017). Based on this shared expression, the fate of this group of cells remains undefined until the late stage of specification. Importantly, any perturbation during the specification period could change the cell fate and collectively contribute to a range of birth defects that may affect the brain, skull, face, and sensory organs (for review see (Thawani and Groves, 2020)). As NPB specification proceeds, the “molecular” separation with the neighboring territories may require DNA methylation/demethylation of the selected genomic regions as well as transcriptional changes. At present, little is known about how DNA methylation/demethylation of NP and NPB is regulated to render some regions of the genome accessible in each lineage, while simultaneously placing the other regions permanently closed.

Folate is an essential vitamin for vertebrates which is taken up by cells through the reduced folate carrier (RFC1, also known as SLC19A1) and the folate receptor alpha FOLR1 (Ratnam et al., 1989; Kur et al., 2014). Both folate deficiency and mutations in genes involved in the folate pathway cause several developmental defects including neural tube and neural crest-related defects in the vertebrate models and humans (Burgoon et al., 2002; Tang et al., 2005; Wilcox et al., 2007; Wehby and Murray, 2010; Li et al., 2011; Lee et al., 2012; Kao et al., 2013; Momb et al., 2013). Inside the cell, folate is required for one-carbon metabolism to transmit methyl groups for the production of S-adenosylmethionine (SAM). It is also required for the methylation of several targets including DNA by DNA methyltransferases (DNMTs). DNA methylation of promoters, enhancers, and transcription start sites is usually linked with gene repression. However, when it is located within gene bodies, it may stimulate elongation of the corresponding transcripts (Buck-Koehntop and Defossez, 2013; Spruijt and Vermeulen, 2014). In contrast, active DNA demethylation involves enzymatic oxidation of 5 mC followed by replacement of its oxidative derivatives with unmodified cytosine. TET (ten–eleven translocation) enzymes (TET1/2/3) oxidize 5 mC to 5-hydroxymethylcytosine (5 hmC), 5-formylcytosine (5fC), and 5-carboxylcytosine (5caC). The last two can then be replaced by the components of base excision repair (BER) machinery to the unmodified cytosine (He et al., 2011; Ito et al., 2011; Maiti and Drohat, 2011; Zhang et al., 2012). Importantly, 5 hm C/5fC/5caC cannot be maintained by the maintenance methylation machinery, so will be passively diluted in dividing cells. It is well-known that DNA methylation plays a major role during early development, and changes in the genomic distribution of cytosine methylation enables the progenitor cells to acquire specific cell fates (Chen et al., 2013; Hore et al., 2016; Hore, 2017; Yu et al., 2018). The role of folate, or the synthetic folic acid, in DNA methylation has been extensively studied in humans, but contradictory roles have been described (Fryer et al., 2009; Ba et al., 2011; Chang et al., 2011; Hoyo et al., 2011). Although some studies suggest that folate/folic acid in the maternal diet may change fetal DNA methylation, no clear association between folate availability and DNA methylation has yet been identified. Thus, studies in the vertebrate models with nonmaternal associated development may shed light on the role of folate deficiency and related changes in DNA methylation during early development.

Here, we use chick embryos to explore the hypothesis that folate is linked to DNA methylation/demethylation during NP and NPB segregation. To test our hypothesis, we characterized the spatiotemporal expression of folate transporters and methylation and hydroxymethylation writers during early development. We used immunohistochemistry to characterize the abundance and distribution of 5mC and 5 hmC at the NP and NPB territories, respectively. Next, we identified the differential methylated regions (DMRs) on RFC1-deficient embryos using reduced representation bisulfite sequencing (RRBS) at a single-base resolution. Finally, to evaluate if RFC1 deficiency affects NPB specification/differentiation, we analyzed the expression of neural and neural plate border markers.

## Results

### 5mC and 5 hmC Abundance and Writers’ Distribution During Early Neural Plate Border Specification

**FIGURE 1:**
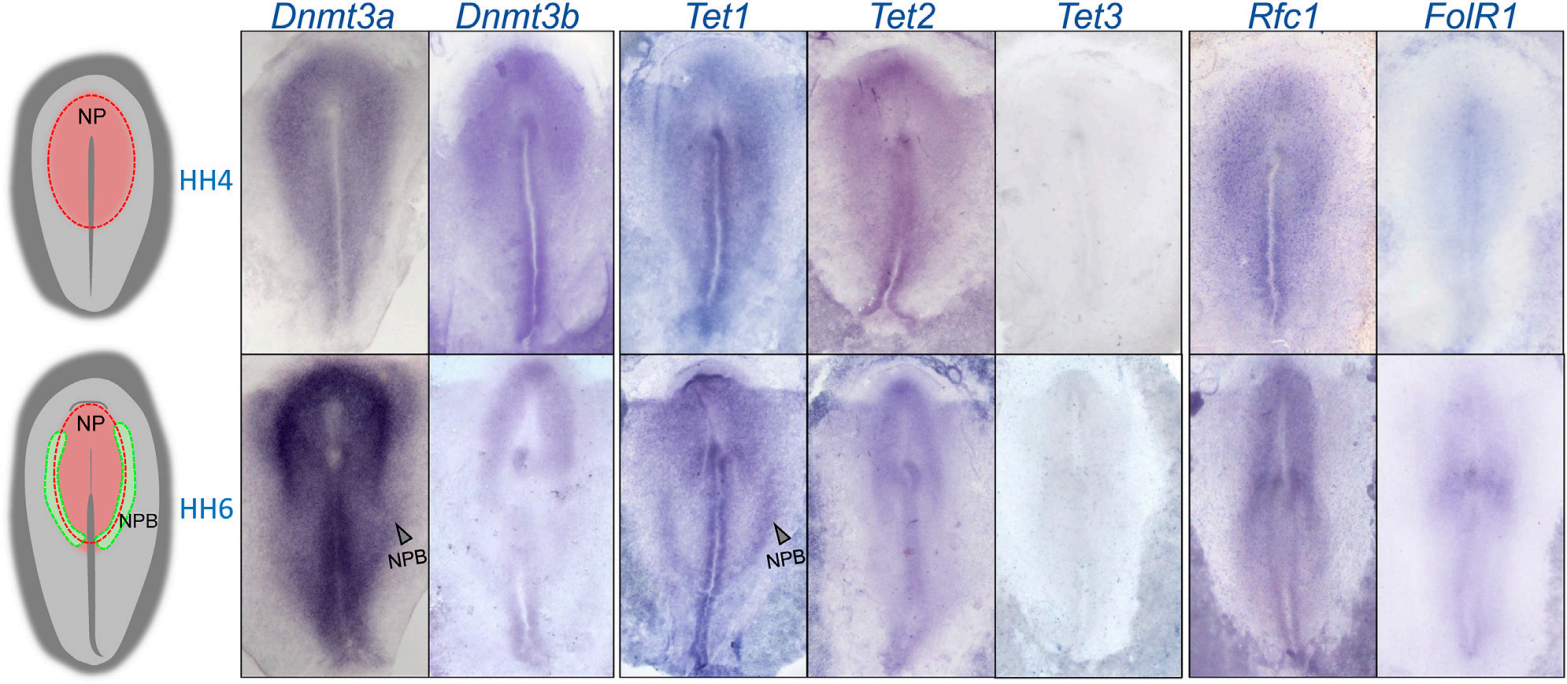
Spatio-temporal expression of 5mC and 5 hm C writers and folate transporters. A rough schematic of a HH4 and HH6 embryos showing the respective positions of the neural plate (NP) and neural plate border (NPB). Whole mount *in situ* hybridization of *Dnmt3a*, *Dnmt3b*, *Tet1*, *Tet2*, *Tet3*, *FolR1*, and *Rfc1* during gastrulation (HH4) and early neurulation (HH6).

To determine the expression patterns of 5mC *de novo* writers (*Dnmt3a* and *Dnmt3b*), 5 hmC writers (*Tet1*, *Tet2,* and *Tet3*) and folate transporters (*Rfc1* and *FolR1*) during NPB specification, we first analyzed their transcript distribution by *in situ* hybridization during the early chick embryo development (Figure 1). The results showed that *Dnmt3a*, *Dnmt3b*, *Tet1-2,* and *Rfc1* genes are expressed at the NP territory during NPB induction (Hamburger and Hamilton stage [HH] 4). However, the expression of *FolR1* was weak and *Tet3* was undetectable. At HH6, when NPB segregation is occurring, both *Dnmt3a* and *Dnmt3b* showed consistent expression in the NP, and in particular, *Dnmt3a* showed a higher expression in the NPB. During this stage, although *Tet3* expression was not detected, we observed a differential distribution of *Tet1* and *Tet2*. Specifically, while *Tet2* is weakly expressed in the NPB, *Tet1* expression is stronger in the posterior NPB. Importantly, the folate transporter *Rfc1* was strongly detected in the NP and moderately in the NPB, while *FolR1* had weaker expression, mostly detected at the location of the future first somite.

**FIGURE 2:**
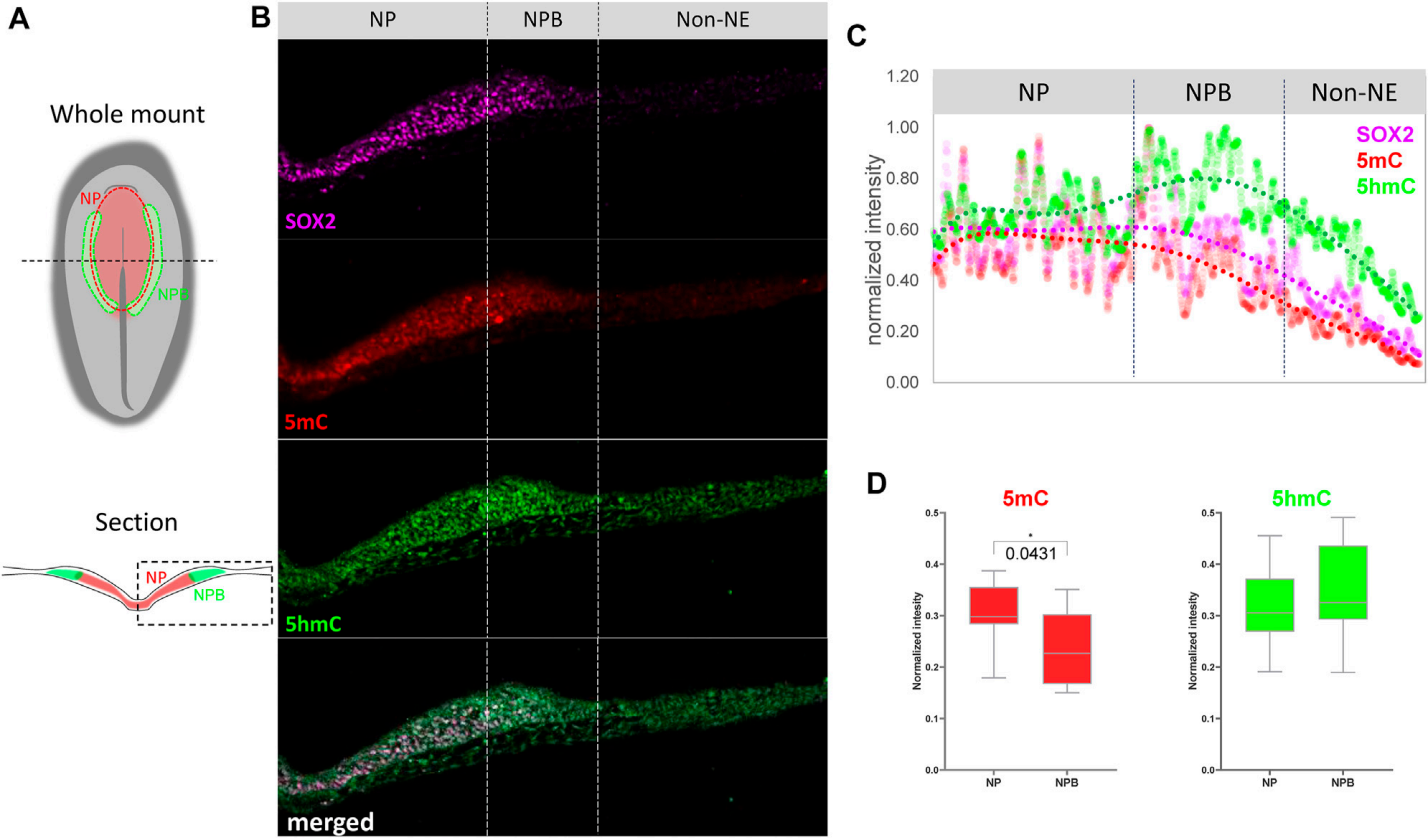
Abundance of 5mC and 5 hm C at the NP and NPB. **(A)** Schematic diagrams of chicken embryo at HH6 showing the respective positions of the neural plate (NP) and neural plate border (NPB). Dashed line in the whole mount indicates level of section. Dashed box in section indicate the area displayed in **(B)** Transverse sections of embryos at HH6 were coimmunostaining for 5mC (red), 5 hmC (green), and SOX2 (magenta). Dashed lines indicate the NP, NPB, and Non-NE regions. See Supplementary Figure S1 for magnification of the NP/NPB boundary. **(C)** Intensity profiles for 5mC (red), 5 hmC (green) and SOX2 (magenta) across the NP, NPB, and Non-NE from medial to lateral. **(D)** Quantification of 5mC and 5 hmC normalized intensity at the nucleus of each cell along the NP and NPB at HH6 (2-5 section/embryo. *n* = 4). Asterisk indicates significance as calculated using Student’s *t*-test. See Supplementary Figure S1 for the negative control of 5mC and 5 hmC immunostaining omitting the primary antibodies.

In order to evaluate if the changes in expression are accompanied by variations in abundance of methylation marks in the NP and NPB, we performed immunohistochemistry against 5mC and 5 hmC on embryos at HH6 (Figures 2A,B; Supplementary Figure S2). To evaluate their abundance, their normalized fluorescence intensity was analyzed according to Roelling *et al.* (2017) (Figure 2C). The expression of SOX2 was used to define NPB territorial extension as it is highly expressed in the NP, but its intensity is lower at the NPB, and is undetectable in the non-neural ectoderm (Non-NE). Similar to the expression of the folate transporters and the methyl-writers and -erasers in Figure 1, the intensity of 5mC was higher in the NP and lower during the transition from the NPB to the Non-NE. In contrast, 5 hmC was detected in the NP with a higher intensity in the NPB. These results correlate with the high expression level of *Dnmt3a* and *Tet1* in this region. Analysis of the relative 5mC and 5 hmC fluorescence in individual cells located at the NP and NPB regions indicated that the intensity of 5mC is higher in the NP (0.3047 ± 0.0606) than the NPB (0.2366 ± 0.0705) (*p* = 0.0431) (Figure 2D). However, there were no significant differences in 5 hmC intensity when comparing the cells located at the NP (0.3194 ± 0.0782) and NPB (0.3475 ± 0,902). This lack of differences could be a result of the variability observed in the individual cells located in the NPB. In conclusion, we observed differential expression of methyl-source, methyl-writers, methyl-erasers, and the relative abundance of 5mC/5 hmC. These results suggest that there could be active turnover of DNA methylation at the NPB, which could be involved in gene regulation in this region.

### Folate Carrier Deficiency Generates Hypomethylated DMRs in the Notch1 Locus and Affects Its Expression

**FIGURE 3:**
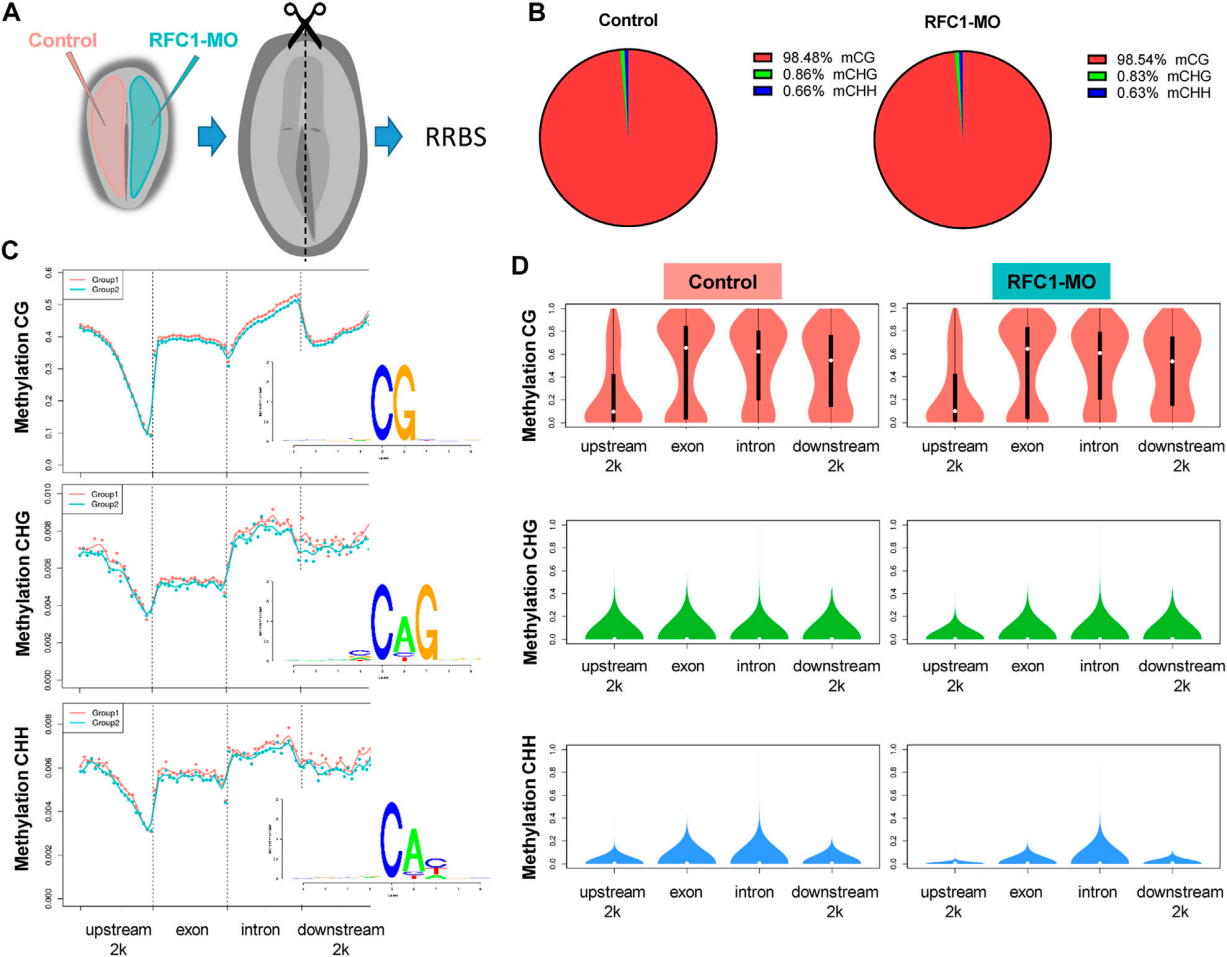
Global analysis of cytosine methylation by reduced representation bisulfate sequencing (RRBS) on folate carrier deficient embryos. **(A)** Schematic representation of loss-of-function experiments performed to generate a folate carrier deficient model. Embryos were bilaterally electroporated with Rfc1 (RFC1-MO) or control (Control) morfolinos, grown until HH7 and genomic DNA was isolated from each side for RRBS sequencing. **(B)** Pie chart analysis of overall cytosine methylation at the three genomic contexts (CG, CHG, and CHH) in control and RFC1-MO treated embryos. **(C)** Comparative analysis of methylation levels in distinct genomic functional elements displayed for CG, CHG, and CHH in control and RFC1-MO treated embryos. Motif occurrence for CG, CHG, and CHH context are shown on the insets for each graph. **(D)** Averages for methylation levels in functional regions for CG, CHG, and CHH in control and RFC1-MO treated embryos. Nonsignificant differences were found in all the global methylation analysis **(B,C,D)** between treatments. upstream2k: 2 Kb upstream of the transcription start site; downstream2k: 2 Kb downstream of the transcription end site.

**FIGURE 4:**
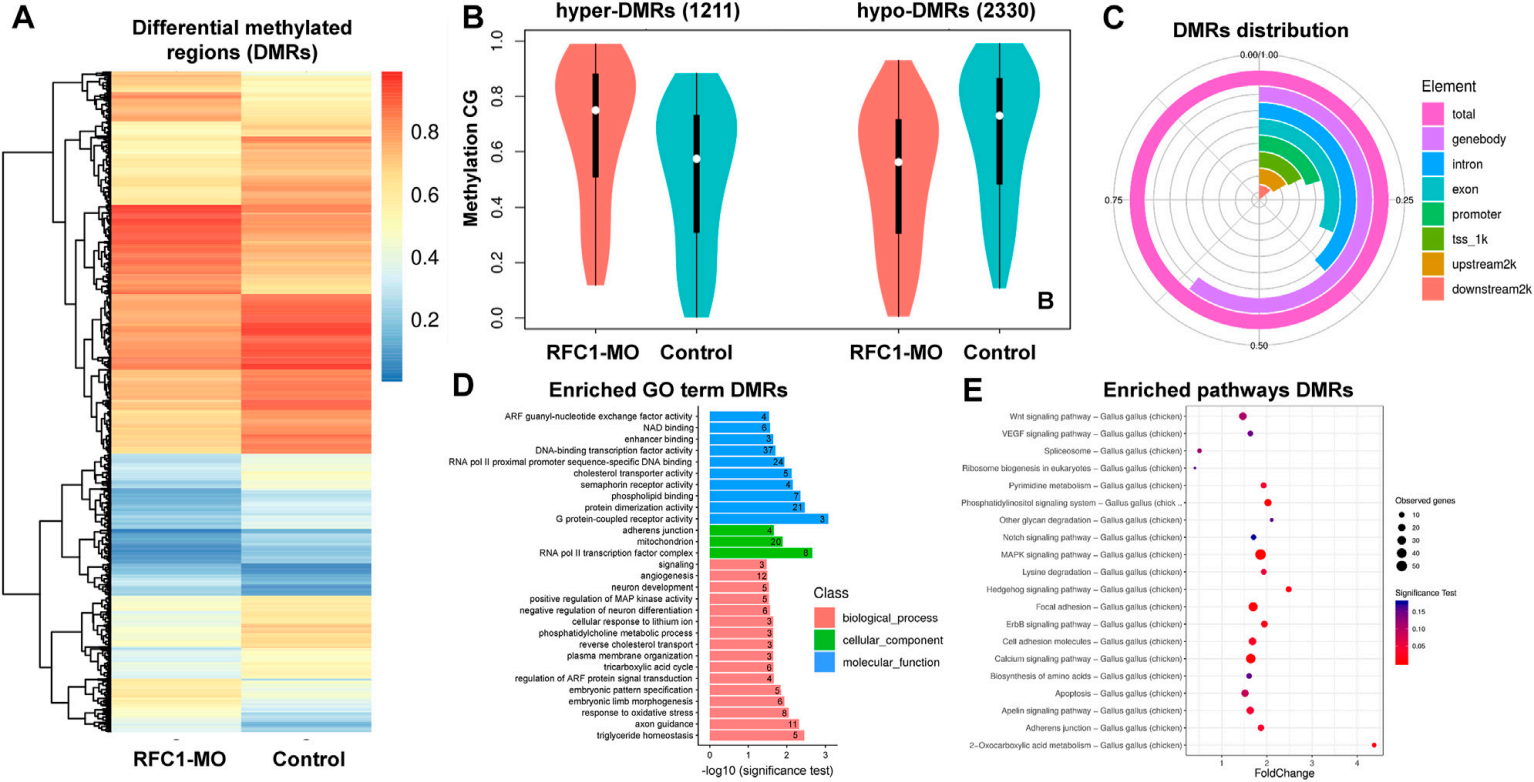
Identification of differentially methylated regions (DMRs) on folate carrier deficient embryos. **(A)** Heatmap of DMRs showing significant differences in CG methylation between the folate deficient (RFC1-MO) and control (Control) embryos. Each raw in the heat map correspond to data point from single region, whereas column correspond to treatments. The branching dendogram correspond to the relationship among data point as determined by clustering using 3,541 DMRs. Hyper- and hypomethylated regions are shown on a continuum from red to blue, respectively. See also Supplementary Table S1. **(B)** Violin boxplot comparing hyper- (1,121) and hypo- (2,330) DMRs between the control and RFC1-MO embryos. **(C)** Genomic distribution of the DMRs. From the 3,541 DMRs, 2,145 were at the gene body, 590 upstream2k, 493 downstream2k, 1,348 in introns, and 712 in promoters. **(D)** Bar plot showing enriched Gene Ontology (GO) terms from genes associated with the DMRs. Numbers inside each bar indicates the total of GO-related genes. The horizontal axis indicates the significance of the test (adjP). See also Supplementary Table S2. **(E)** Scatter plot showing enriched KEGG pathways from genes associated with the DMRs. The vertical axis represents the pathway and the horizontal axis represent the fold change. The size of the dots indicates the number of affected genes in the pathway and the color corresponds to a different *p*-value range. See also Supplementary Table S3. DMRs analysis was performed by using metilene software using a double statistical test (MWU-test and 2D KS-test) followed by multiple test correction (adj.*p*-value). GO enrichment and KEGG enrichment significance was evaluated by raw*P* (Hypergeometric test *p*-value) followed by correct *p*-value (adjP).

Since our results identified the dynamic expression of DNA methylases and demethylases, we evaluated how perturbing the methyl-source would alter the DNA methylation status during NPB restriction and fate specification. To address this, we disrupted the folate intake into the cells by affecting the expression of *Rfc1* during NPB specification using a previously validated morpholino (RFC1-MO) (Alata Jimenez et al., 2018). To this end, embryos were unilaterally electroporated with a control morpholino on one side and the RFC1-MO on the alternate side. At HH7, the embryos were bisected and differential methylation was analyzed using the RRBS-seq (Figure 3A). We did not observe significant differences in global DNA methylation between the control sides and those lacking RFC1 in major gene-associated regions (upstream 2kb, exons, introns, and downstream 2 kb) at the three possible methylation sites (CG, CHG, and CHH) (Figures 3B–D). Consistent with this notion, immunostaining for 5mC and 5 hmC shows no significant difference in the normalized intensity for 5mC and 5 hmC analyzed at the NP and NPB in the RFC1-MO and control-MO injected embryos (Supplementary Figure S3). However, 3,541 differentially methylated regions (DMRs), of which 2,374 were associated to genes, were identified. Specifically, 1,211 hypermethylated and 2,330 hypomethylated regions were identified in the RFC1-MO treated tissues compared to the control (Figures 4A,B. Supplementary Table S1). Most of these DMRs were located in the gene body (60%, 2,145 DMRs) and to a lesser extent in the promoter region (16%, 590 DMRs) (Figure 4C).

Gene Ontology (GO) analysis of DMRs showed an enrichment in genes involved in biological processes including DNA-binding transcription factors (37 genes), RNA pol II proximal promoter sequence-specific DNA binding (24 genes), RNA pol II transcription factor complex (eight genes), embryonic pattern specification (five genes), factors related with neuron development (five genes), negative regulation of neuron differentiation (six genes), and embryonic pattern specification (five genes), among others (Figure 4D. Supplementary Table S2). Additionally, WNT, Hedgehog, MAPK, and NOTCH signaling pathways were enriched and had associated DMRs (Figure 4E. Supplementary Table S3).

**FIGURE 5:**
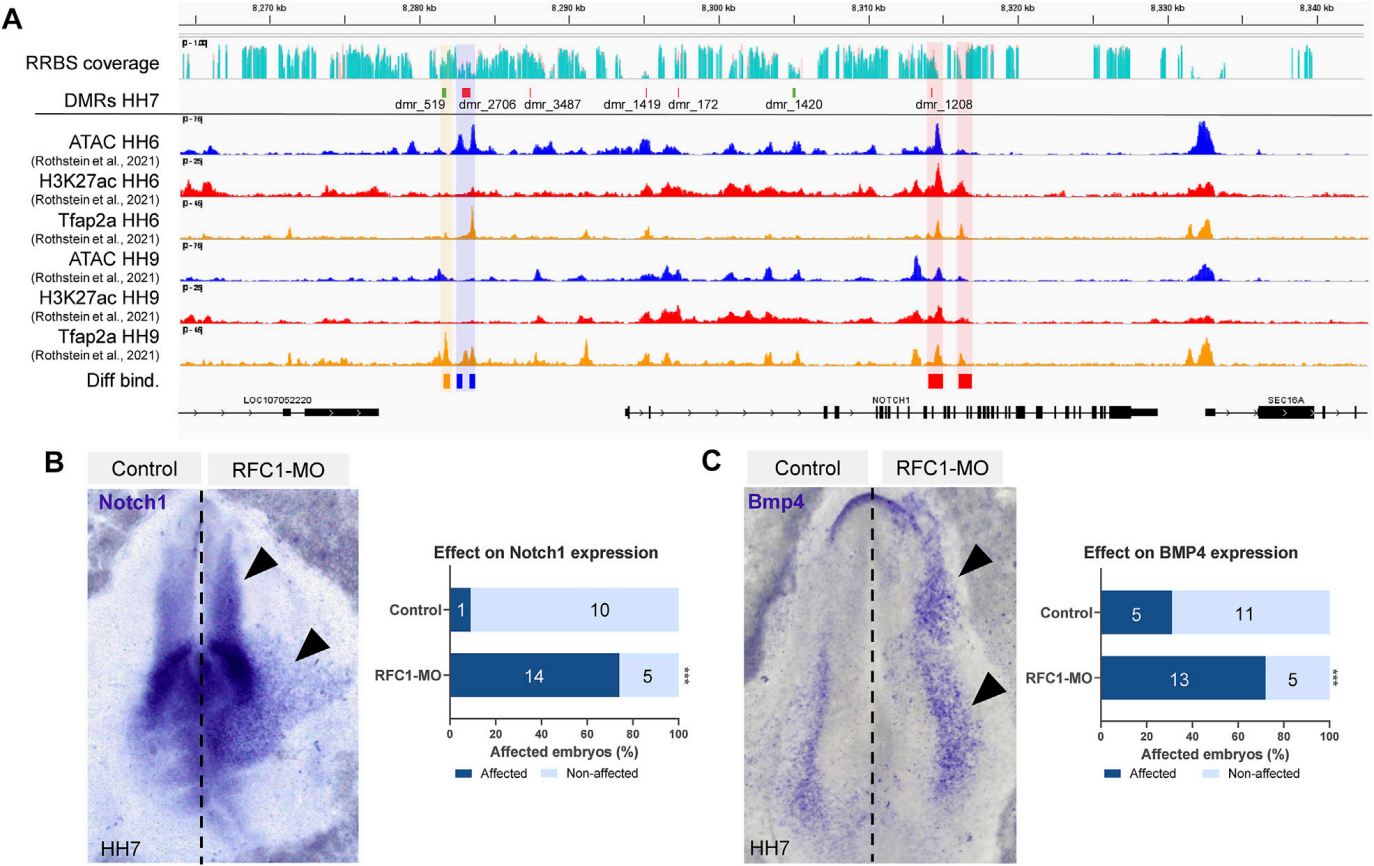
Folate carrier deficiency generated DMRs at the Notch1 locus and affect its expression. **(A)** IGV visualization of the *Notch1* locus and its vicinity, RRBS read coverage (Control in turquoise and RFC1-MO in orange), hyper (green) and hypo (red) methylated DMRs at HH7 embryos. ATAC-seq, H3K27ac, and TFAP2A CUT&RUN profiles at HH6 and HH9 (data obtained from Rothstein *et al.* (2020)). Three DMRs are found upstream of the *Notch1* gene, whereas four are located in the gene body. Differentially accessible elements (shaded in blue), H3K27ac abundance (shaded in red), or Tfap2A binding (shaded in orange) comparing HH6 and HH9 were determined by DiffBind using a negative binomial distribution model (FDR <0.1). Whole-mount *in situ* hybridization of *Notch1* **(B)** and *Bmp4* **(C)** in the bilaterally electroporated embryos injected with control or RFC1 morpholinos. Black arrowhead pointed out increased and ectopic expression in the treated side. Bar graphs represent the quantitation of embryos following RFC1-MO presenting increased and ectopic expression for *Notch1* or *Bmp4* (affected) and comparing with the control-MO treated embryos. Numbers in the graph represent the number of analyzed embryos. Statistical analysis were performed by the contingency table followed by Fisher’s exact test (***: *p* = 0.0001).

DMRs associated with genes involved in transcription and embryo patterning is consistent with a role of DNA methylation during early territorial restriction. Particularly, we identified that *Notch1* locus contained four DMRs in the gene body (dmr_1419, 172, 1420, and 1208) and three upstream (dmr_3487, 2706, and 519) (Figure 5A). Interestingly, we observed that several of these DMRs were located in the genomic regions that are affiliated with variable chromatin accessibility (ATAC-seq data), contain the enhancer mark H3K27ac, and/or are targets of the pioneer factor TFAP2A (data obtained from Rothstein & Simoes-Costa (Rothstein and Simoes-Costa, 2020)). Particularly, out of the seven regions that have DMRs near the *Notch1* gene, dmr_2706 is flanked by two open chromatin regions (shaded in blue in Figure 5A) that experience significant changes in their accessibility as development proceeds from HH6 to HH9 (*p*-value: 1.62E-08, FDR: 3.57E-05; *p*-value: 4.64E-08, FDR: 6.62E-05). In our samples, the dmr_2,706 remains hypomethylated, thus suggesting that this chromatin region may be open in our RFC1-deficient embryos. Interestingly, the binding of the pioneer transcription factor TFAP2A, which has been implicated in epigenomic remodeling during the neural crest specification (Chang et al., 2011) presented significant increase (*p* value: 0.00755, FDR: 0.105) in the binding at HH9 to a region located close to dmr_519 upstream to the *Notch1* gene (shaded in orange on Figure 5A). Moreover, differential changes in H3K27ac abundance are observed in two regions (*p* value: 1.62E-08, FDR: 3.57E-05; *p*-value: 4.64E-08, FDR: 6.62E-05) found in the proximity of dmr_1208 located in an intron of the *Notch1* gene (shaded in red on Figure 5A).

The NOTCH1 pathway has been described as a key factor in the NP/NPB segregation in vertebrates. Specifically, the NOTCH1 interaction with DELTA1 regulates *BMP4* expression in the *epidermis*, and is thus indirectly required for NPB induction between the non-NE-NP boundary (Endo et al., 2002; Glavic et al., 2004; Nagatomo and Hashimoto, 2007). Based on this precedent, we evaluated the effect of RFC1 deficiency on *Notch1* and *Bmp4* expression. Our results revealed that loss of *Rfc1* increased and expanded the expression of both *Notch1* and *Bmp4* compared to the contralateral uninjected side or injected with Control-MO (Figures 5B,C).

Taking together, these results indicate a correlation between DNA methylation at the specific genomic regions and the expression and regulation of *Notch1* gene during development. However, further studies are needed to confirm a mechanistic causal relationship.

### Folate Deficiency Enhances the Bipotentiality of Neural Plate Border Cells

**FIGURE 6:**
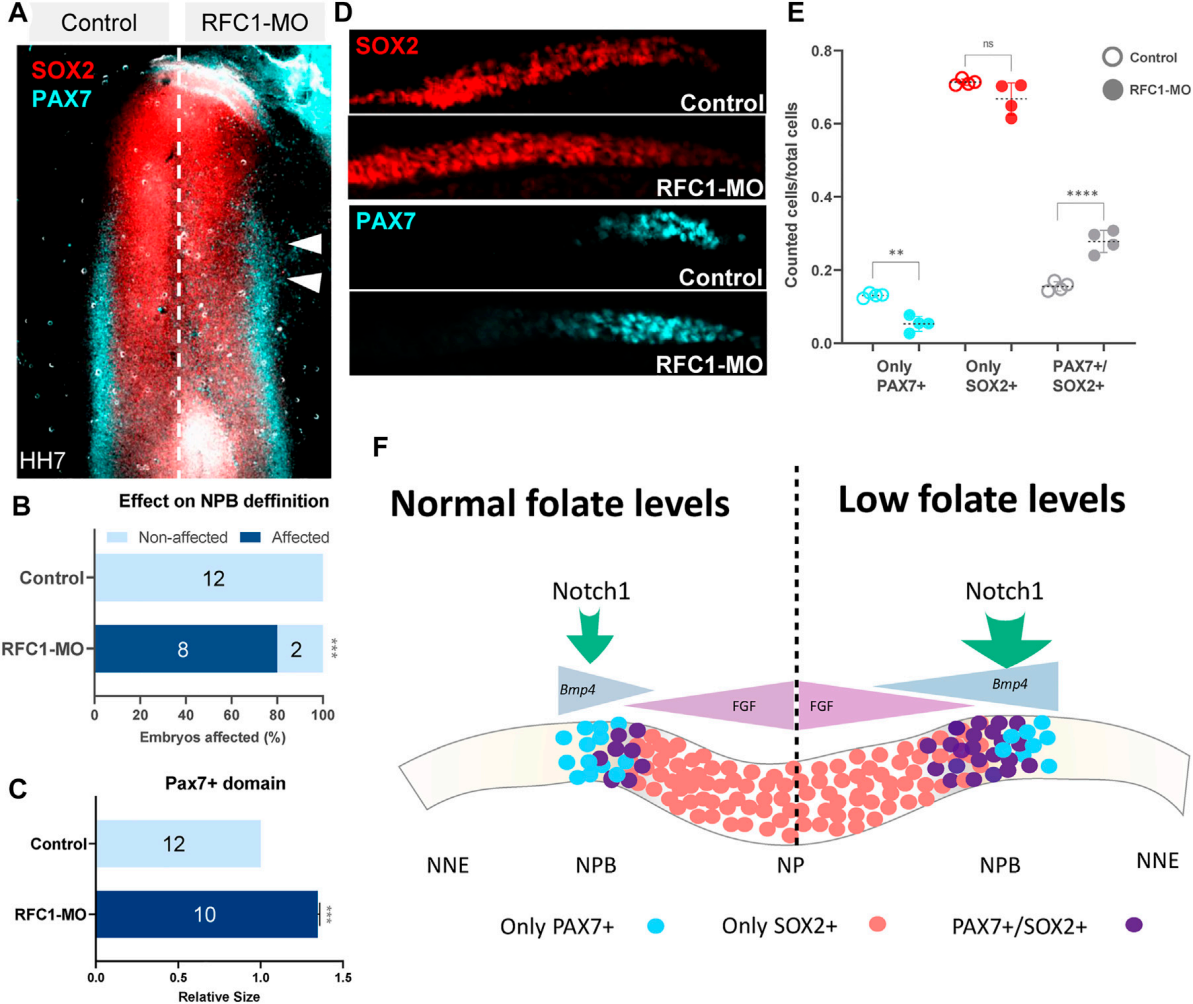
Folate carrier deficiency enhances the potentiality of the NPB cells. **(A)** Whole mount immunohistochemistry of PAX7 (neural plate border marker) and SOX2 (neural marker) in the RFC1-MO and control-MO treated embryos. White arrowhead pointed out ectopic expansion of PAX7+ cells. **(B)** Quantitation of embryos electroporated with RFC1-MO (*n* = 10) presenting ectopic expression of PAX7+ cells and comparing with control-MO (*n* = 10) treated embryos. **(C)** Quantification of the relative size of PAX7+ cells domain observed in **(D) (D)** Transversal sections of the treated embryos followed by IHC against SOX2 and PAX7 markers. Orientation of control sides has been flipped for comparison. **(E)** Scatterplot of quantification of cells that exclusively expressing PAX7+, SOX2+, or coexpressing both PAX7+/SOX2+ on RFC1-MO and control embryos. Statistical analysis in B chart was performed by the contingency table followed by Fisher’s exact test (***: *p* = 0.0001). For **(E)**, Ordinary one-way ANOVA followed by Tukey’s multiple comparison test (**: *p* = 0.003, ****: *p* < 0.0001). **(F)** Schematic proposed model: Low levels of folate during the early stages of NPB development affect DNA methylation and expression of *Notch1*, that leads to an increased and expanded *Bmp4* expression. As consequence, a higher number of progenitors in a “primed” state are maintained due to the coexpression of both the neural and neural plate border markers at their territorial boundaries.

Folate deficiency alters neural crest specification, migration, and even differentiation (Burgoon et al., 2002; Tang et al., 2005; Li et al., 2011; Momb et al., 2013; Wahl et al., 2015; Alata Jimenez et al., 2018). However, the role of folate and DNA methylation during NP and NPB segregation is unclear. Considering that the proteins driving DNA methylation (*Dnmt*s, *Tet*s, and *Rfc1*) are expressed early in development, and due to the presence of DMRs in the *Notch1* gene in *Rfc1*-deficient embryos, we evaluated the role of folate on NPB specification. As the loss of *Rfc1* increased *Notch1* and *Bmp4* expression, we evaluated how its loss impacted the fate of the progenitors residing in the NPB. To this end, whole-mount immunohistochemistry against SOX2 (NP marker) and PAX7 (NPB marker) revealed that the loss of *Rfc1* also expanded the NPB region compared to the control side (Figure 6A). We observed that 80% of the embryos exhibited an expansion of the NPB (*p*-value = 0.001. Figure 6B), which was expanded by 1.35 fold compared to the control side (*p*-value<0.001. Figures 6C,D).

Previous work identified an overlap in the expression of transcription factors associated with diverse lineages in the single NPB cells (Roellig et al., 2017). Based on this prior work, we analyzed the level of PAX7 and SOX2 coexpression in *Rfc1*-deficient embryos (Figure 6E). We observed a significant reduction in the number of cells that exclusively expressed PAX7 compared to the control side (*p* value = 0.003). However, no differences were observed in the SOX2+ cells (*p* value = 0.134). Interestingly, a significant increase in the PAX7+/SOX2+ double positive cells (*p* value<0.0001) was observed when compared to the control side. As the increase in the double positive cells (PAX7+/SOX2+) was greater than the reduction in the PAX7+ only cells, thus an overall increase in the size of PAX7 domain is observed (Figure 6E). These data suggest that folate deficiency expanded the NPB territory by enhancing the cellular potential of those progenitor cells. Of note, although the loss of RCF1 expanded *Bmp4* expression and increased the amount of PAX7+/SOX2+ cells, none of these genes had DMRs enriched for the open chromatin/H3K27ac/TFAP2A in their vicinities (Supplementary Figure S4), suggesting an indirect effect possibly mediated by the dysregulation of the *Notch1* gene.

Taking together our findings, we suggest a hypothetical model where the lack of RCF1 affects DNA methylation at specific regions which correlated with the dysregulation of pathways involved in early development such as *Notch1*-*Bmp4* signaling. These changes may affect the potential of the progenitor residing in the juncture of the NP/NPB territories, thus maintaining them in a more primed state (Figure 6F).

## Discussion

Territorial specification and fate restriction from a stem cell into a specialized cell type often involves several epigenetic changes, where DNA methylation/demethylation plays a critical role. During early development, the progenitors residing in the NPB segregate from the NP and non-NE and maintain the potency to differentiate into neural, neural crest, placodal, and epidermal cells (Roellig et al., 2017). These intermingled lineage states, achieved by the expression of mixed-lineage transcriptional programs (known as “priming”), have also been described during stem cell fate determination in the hematopoietic system (Hu et al., 1997; Laslo et al., 2006; Olsson et al., 2016). In mammals, *de novo* methylation leads to a global gain of 5mC *in vivo* during the transition toward the primed pluripotent state (Seisenberger et al., 2012; Smith et al., 2012; Auclair et al., 2014; Wang et al., 2014). A similar event occurs during the transition from naïve to primed states in the embryonic stem cells (ESCs) *in vitro* (Ficz et al., 2013; Habibi et al., 2013; Leitch et al., 2013; Takashima et al., 2015; von Meyenn et al., 2016). Here, we demonstrated that DNA methylation turnover, exerted by DNMT3s and TETs activities, are differentially required during NP and NPB specification when the embryo goes from gastrulation to neurulation. The folate transporter RFC1 appears to be the major source of SAM playing a crucial role modulating 5mC/5 hmC abundance in specific genomic regions during NPB specification. It is interesting to note that high abundance and variation of the 5 hmC mark at the NPB may be linked to an increased and/or retained potentiality (Blaschke et al., 2013; Chen et al., 2013; Yu et al., 2018), as well as reprograming of the epigenetic memory (Monfort and Wutz, 2013; Hore et al., 2016; Hore, 2017). Moreover, the level of 5mC/5 hmC at the NPB may suggest a dynamic system, with a constant turnover of cytosine modifications that could lead to heterogeneous epigenetic states that might affect gene expression and cell fates. In agreement with this notion, a recent study demonstrated that DNA methylation heterogeneity arises during the transition from naïve-to-primed pluripotency (Rulands et al., 2018), indicating that dynamic changes in DNA methylation might influence early cell fate decisions during the early embryo development.

As a methyl donor, the lack of folate may alter the global abundance of DNA methylation. However, we did not detect changes in global methylation at the canonical (CpG) or noncanonical methylation (CHG and CHH) by RRBS analysis on embryos where the expression of RFC1was reduced. Among the noncanonical methylations, CA methylation was the most abundant mark detected in our study. Importantly, CA methylation is mostly located at the promoters (Geng et al., 2018), and has a negative correlation with gene expression (Guo et al., 2014; Price et al., 2019). Although CA methylation is affected by folate deficiency (Geng et al., 2018) and has been described to play a critical role in other cellular contexts (Ziller et al., 2011; Guo et al., 2014; Price et al., 2019), further experiments will be required to define its specific participation in the NPB development.

The role of folate/folic acid in DNA methylation has been extensively studied in human, but contradictory effects have been described (Fryer et al., 2009; Ba et al., 2011; Chang et al., 2011; Hoyo et al., 2011). Past studies have demonstrated that folate deficiency/supplementation does not always alter global DNA methylation (Steegers-Theunissen et al., 2009; Chang et al., 2011; Crider et al., 2011; Hoyo et al., 2011; Amarasekera et al., 2014; Tobi et al., 2014; Mahajan et al., 2019), but it certainly affects DNA methylation in certain tissues (Chang et al., 2011; Mahajan et al., 2019), population of cells (Crider et al., 2011), or specific genes (Steegers-Theunissen et al., 2009; Hoyo et al., 2011; Amarasekera et al., 2014; Irwin et al., 2019). In this sense, we observed that RFC1 deficiency generates DMRs throughout the genome, mostly located at the body of specific genes. Interestingly, although a higher number of hypomethylated DMRs were detected, we also evidenced several hypermethylated DMRs throughout the genomes. This sort of feature has also been observed in several maternal studies (Tobi et al., 2014; Joubert et al., 2016; Irwin et al., 2019; Madrid et al., 2020), suggesting that folate deficiency/supplementation affects methylation status in a heterogeneous way.

Folate deficiency is associated with defects in the neural development due to the dysregulation of key genes implicated in their differentiation (Joubert et al., 2016; Geng et al., 2018). Here, we observed DMRs in several genes associated with the neuronal development and embryonic patterning as well as epigenetic processes like chromatin binding. In this sense, the folate, as the main source for methylation, could have a pivotal role on the regulation of well-characterized genes implicated in DNA and histone methylation during NPB specification and neural crest development (Strobl-Mazzulla et al., 2010; Hu et al., 2012; Hu et al., 2014; Rogers and Nie, 2018; Rothstein and Simoes-Costa, 2020).

The specification of the NPB requires interaction of BMPs, FGFs, and WNTs (Basch et al., 2000; Murdoch et al., 2010; Murdoch et al., 2012; Milet et al., 2013), allowing the molecular segregation of the NP and non-NE tissues. In addition, *Notch1* participates in the induction of the NPB by regulating the levels of *Bmp4* and establishing an adequate gradient during the formation of this territory (Endo et al., 2002; Glavic et al., 2004; Nagatomo and Hashimoto, 2007). In that sense, we observed that RFC1-deficiency generates seven DMRs in the *Notch1* gene and its vicinity. Importantly, several of these DMRs are located in regions where chromatin accessibility changes, that are targeted by the pioneer factor TFAP2A or contain the active histone mark H3K27ac at the time of NPB specification (Rothstein and Simoes-Costa, 2020). This is consistent with our observation that folate deficiency expanded the *Notch1* and *Bmp4* expression. Additionally, it correlates with the well-described role of *Notch1*-*Bmp4* pathway to induce the generation of neural crest cells in the ectopic regions (Liem et al., 1995; Endo et al., 2002; Patthey et al., 2009; Steventon et al., 2009; Piacentino and Bronner, 2018). It is important to mention that since RFC1 deficiency may alter SAM availability, other methylation defects (i.e., histone methylation) may also disrupt this pathway.

Our RFC1-deficient model showed that the NPB territory, evidenced by PAX7 expression, was expanded in the treated embryos. However, although this expansion was not due to an extension of the neural territory, a significant number of cells coexpress both SOX2 and PAX7 markers. NPB cells are a “multi-fated” cell population that is capable of coexpressing markers from different lineages (Lignell et al., 2017; Roellig et al., 2017; Williams et al., 2019). Small changes in the transcription factor levels can bias cells, thus allowing them to exit from a “primed” state toward a specific cell fate (Roellig et al., 2017). Our data suggested that RFC1 deficiency is able to maintain or expand the primed state of the progenitor cells residing at the boundaries of NP and NPB. Our results are in agreement with the observation that dynamic oscillations in DNA methylation generate epigenetic heterogeneity during development (Rulands et al., 2018). Our model proposes that these epigenetic changes may influence the cell fate decisions at the NP/NPB boundaries. Thus, NPB cells appear to be highly regulated cells that respond to epigenetic and transcriptional modifications. Single-cell methylation analysis and epigenetic manipulations will shed light on how methylation variability may play a central role in promoting transcriptional heterogeneity at the NPB cells with the attendant consequences for lineage fate decisions.

## Materials and Methods

### Embryos

Fertile chicken eggs were purchased from commercial sources and incubated at 38°C until the embryos reached a desired stage. Chicken embryos were collected and staged according to the criteria of Hamburger and Hamilton (Hamburger and Hamilton, 1992). For *in situ* hybridization, the embryos were fixed overnight at 4°C in PBS–0.1% tween (PBS-t) (pH 7.4) containing 4% paraformaldehyde (PFA), dehydrated, and stored in methanol. For immunochemistry, the embryos were fixed for 20 min in 4% PFA in PBS-t and processed immediately.

### Electroporation of Morpholino and Vectors

We used a previously tested *Rfc1* antisense morpholino (RFC1-MO) (Alata Jimenez et al., 2018) at the boundary of exon–intron in order to block the splicing and to lead to a frameshift and premature stop. Injection of FITC-tagged morpholino (0.75 mM RFC1-MO and 0.75 mM Control-MO) plus 300ng/ul of plasmid DNA (used as carrier) were performed by air pressure using a glass micropipette and targeting the half side of neurula stage embryos. Electroporation was made with five 50 ms pulses of 5.2 V, with intervals of 100 ms between each pulse. The embryos were cultured in 0.75 ml of albumen in tissue culture dishes and incubated at 38°C until the desired stages were obtained. All the embryos were screened prior to further analysis. Those embryos with weak and/or patchy electroporation or with strong morphological abnormalities were discarded.

### *In Situ* Hybridization (ISH)

Whole-mount ISH was carried out as described previously (Kee and Bronner-Fraser, 2001). Digoxigenin-labelled probes were synthesized from linearized vectors containing partial full-length cDNAs of *FolR1*, *Rfc1*, *Dnmt3a*, *Dnmt3b*, *Tet1*, *Tet2*, *Tet3*, *Bmp4m* and *Notch1*. Hybridized probes were detected using an alkaline phosphatase-conjugated antidigoxigenin antibody (Roche, 1:200) in the presence of NBT/BCIP substrate (Roche). Embryos were photographed as a whole-mount using a ZEISS SteREO Discovery V20 Stereomicroscope (Axiocam 512 color) and Carl ZEISS ZEN2 (blue edition) software.

### Cryosectioning

For the histological analysis, embryos were incubated in 5% sucrose (in PBS) for 2 h at room temperature and subsequently transferred to 15% sucrose and incubated overnight at 4°C. After that, the embryos were transferred and incubated in 7.5% gelatin in 15% sucrose for 4 h at 37°C. Then, they were frozen with liquid nitrogen and immediately stored at −80°C for cryosectioning. Transverse section of 10-15um were obtained and used for immunostaining.

### Immunohistochemistry

Embryos were fixed in 4% PFA/Phosphate Buffer (PB) for 20 min at room temperature. The embryos or sections were washed in TBS with 0.5% Triton (TBS-T) and subsequently blocked with 5% FBS in TBS-T for 3 h at RT. The embryos or sections were incubated in primary antibody solution at 4°C fortwo days. The primary antibody used were mouse monoclonal anti-5mC (Abcam, ab10805, 1:500), rabbit polyclonal anti-5hmC (Active Motif, Cat Nº 39791, 1:500), goat polyclonal anti-hSOX2 (Santa Cruz Biotech; Y-17; 1:500, for Figure 2A), rabbit monoclonal anti-SOX2 (Abcam, ab92494, 1:1,000; for Figures 6C,F), and anti-PAX7 IgG1 (Developmental Studies Hybridoma Bank, 1:10). The secondary antibodies used were donkey anti-rabbit 594, donkey anti-mouse 488, donkey anti-goat 647, goat anti-rabbit 594, and goat anti-mouse IgG1 647 (all from Molecular Probes, 1:500). After several washes in TBS-T, the embryos and sections were mounted and imaged by using Carl ZEISS Axio observer 7 inverted microscope (Axio observer Colibri 7, Axiocam 305 color, Axiocam 503 mono) and Carl ZEISS ZEN2 (blue edition) software. The negative controls omitting the primary antibodies (anti-5mC and anti-5hmC) fails to detect any specific mark (see Supplementary Figure S1).

### Quantification of 5 hmC and 5mC Intensities

Measurements of 5 hmC and 5mC were performed by using the ZEN 3.0 blue edition, briefly: Zeiss. czi files intensities for single cell analysis (Figure 2C) was measured by placing a fixed-sized oval (46.09 μm^2^) on cells throughout the NP and NPB. In order to avoid any artifact from the cytoplasm, DAPI staining was performed to locate the oval at the nucleus. Background intensities were measured and a reference area was defined by a fixed-sized oval (oval of 167.61 μm^2^ in a closed region to the primitive streak for 5mC and the middle part of the NPB for 5 hmC). Additionally, the intensity of the background and reference area was measured for each section. In Excel, background was subtracted from the marks and the reference area. For each marker, intensity was normalized to intensity reference area (experimental intensity/reference intensity). Values of all cells per embryo (2-5 sections/embryo, n = 4) were averaged and the significance were calculated using Student’s t-test in Prism 9 Graphpad.

### RRBS-Seq

Genomic DNA from two independent replicates composed by ∼18 electropored embryos with Rfc1-MO and Control-MO each one was extracted by using PurelinkTM Genomic DNA Mini Kit (Invitrogen) following the manufacture’s instruction. The genomic DNA were sent to CD Genomics for library construction, sequencing, and bioinformatics analysis. Briefly, the DNA samples were digested with methylation-insensitive restriction enzyme MspI. DNA fragments were end-repaired adding A tail and ligated with the sequencing linker, where all cytosine are methylates. DNA fragments from 150–300 bp were selected and treated with bisulfite by using EZ DNA Methylation Gold Kit (Zymo Research). Finally, PCR amplification was performed to obtain the final DNA library and the sequencing was performed with Illumina HiSeq PE150. Bisulfite conversions were >99.5% for all the samples. Basic statistic of the quality of the raw reads were performed with FastQC tool (http://www.bioinformatics.babraham.ac.uk/projects/fastq). Then, sequencing adapters and low-quality data (sequence in which N’s content is greater than 5% of the total length of the sequence) were removed by using Trimmomatic software (V0.36) (Bolger et al., 2014). The obtained sequences were mapped to the galGal5 genome reference with BSMAP software (Xi and Li, 2009). The statistic information of the alignment is collected, only the unique mapped reads were kept for the following analysis and only the methylated cytosine with sequence depth coverage of at least 5 were used. The methylation levels of individual cytosines were calculated as the ratio of the sequenced depth of the ascertained methylated CpG cytosines to the total sequenced depth of the individual CpG cytosines. The software metilene (V0.2–7) was used to identify DMR (differentially methylated regions) by a binary segmentation algorithm combined with a two-dimensional statistical test (Jühling et al., 2016).

Gene Ontology (referred to as GO, http://www.geneontology.org/) enrichment analysis of the DMR-related genes was applied to uncover biological processes of interest, we chose to deem pathways with a Q value <0.05 as significantly enriched with the DMR-related genes. Based on the results of the DMR annotation and the database of Kyoto Encyclopedia of Genes and Genomes (KEGG) (Kanehisa et al., 2008), functional enrichment analysis was performed on genes whose gene body and its upstream and downstream regions (upstream 2 k, gene body, and downstream 2 k) overlap with DMR.

## Data Availability

The datasets presented in this study can be found in online repositories. The names of the repository/repositories and accession number(s) can be found in the article/Supplementary Material.
