# Supplementary figures and images for: Folate Carrier Deficiency Drives Differential Methylation and Enhanced Cellular Potency in the Neural Plate Border
Source: Front Cell Dev Biol. 2022 Jul 13;10:834625. doi: 10.3389/fcell.2022.834625 (PMC9326018; doi:10.3389/fcell.2022.834625)

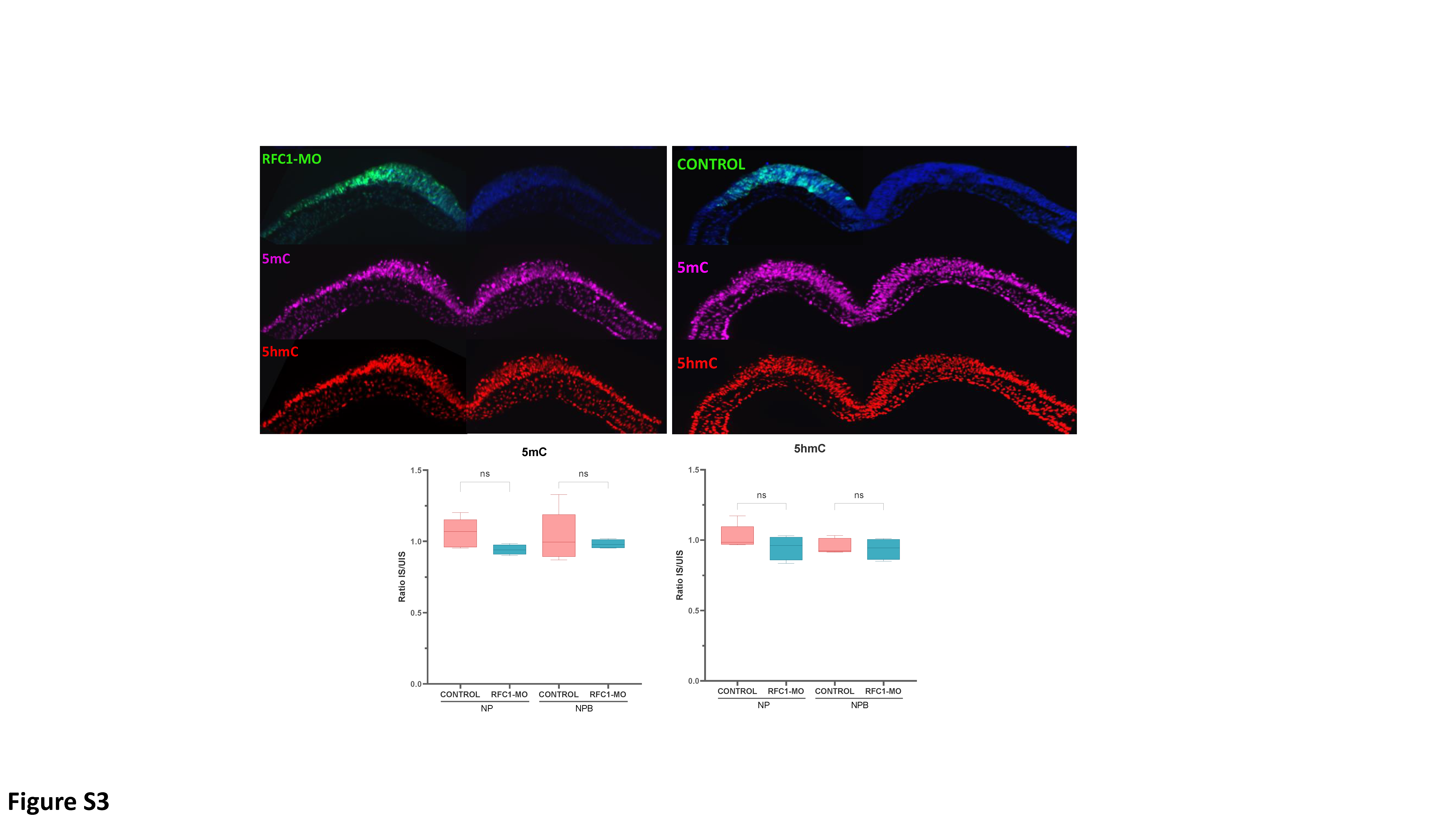

Supplement: Supplementary file 1 [file Image3.TIFF]

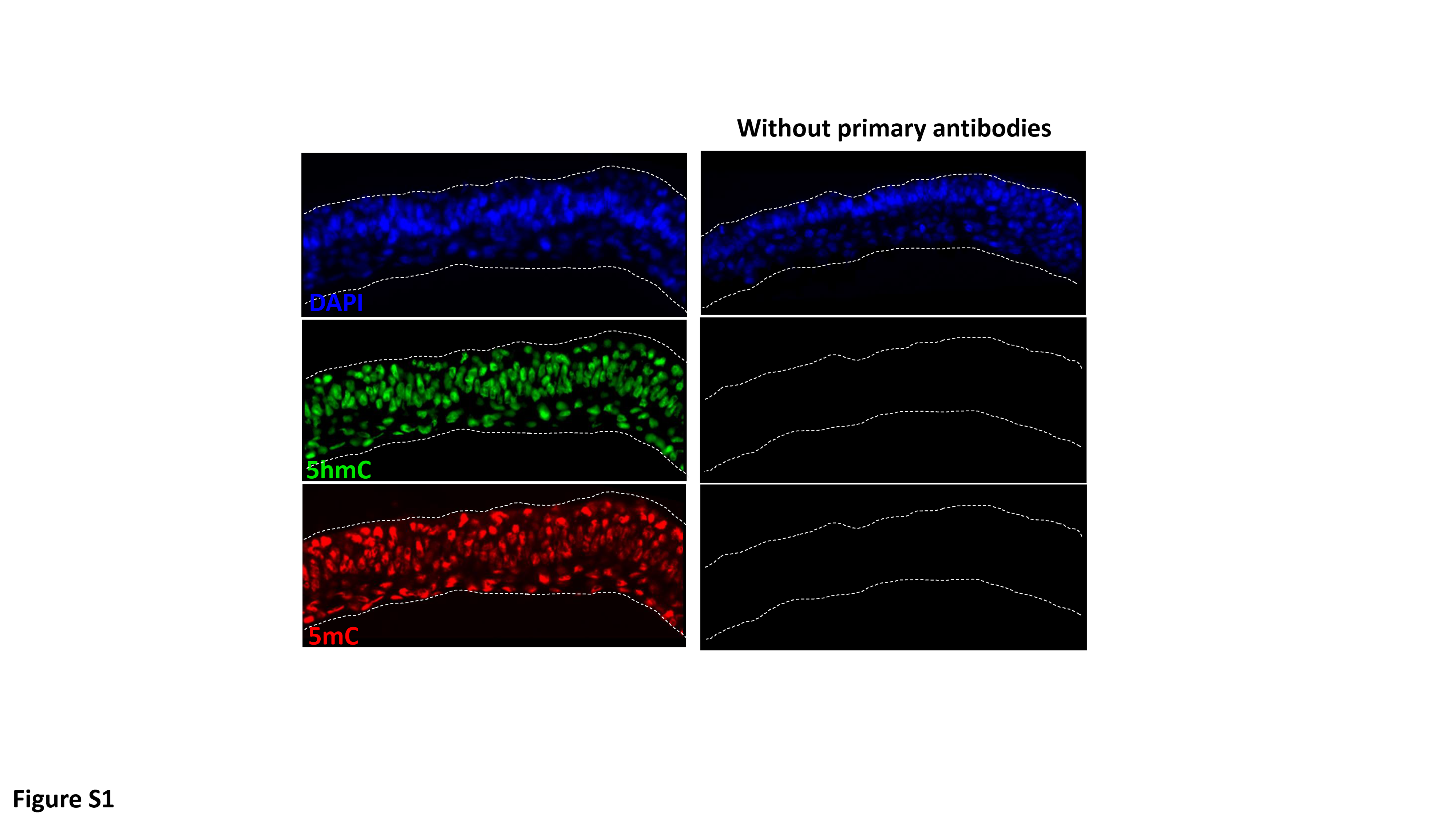

Supplement: Supplementary file 3 [file Image1.TIFF]

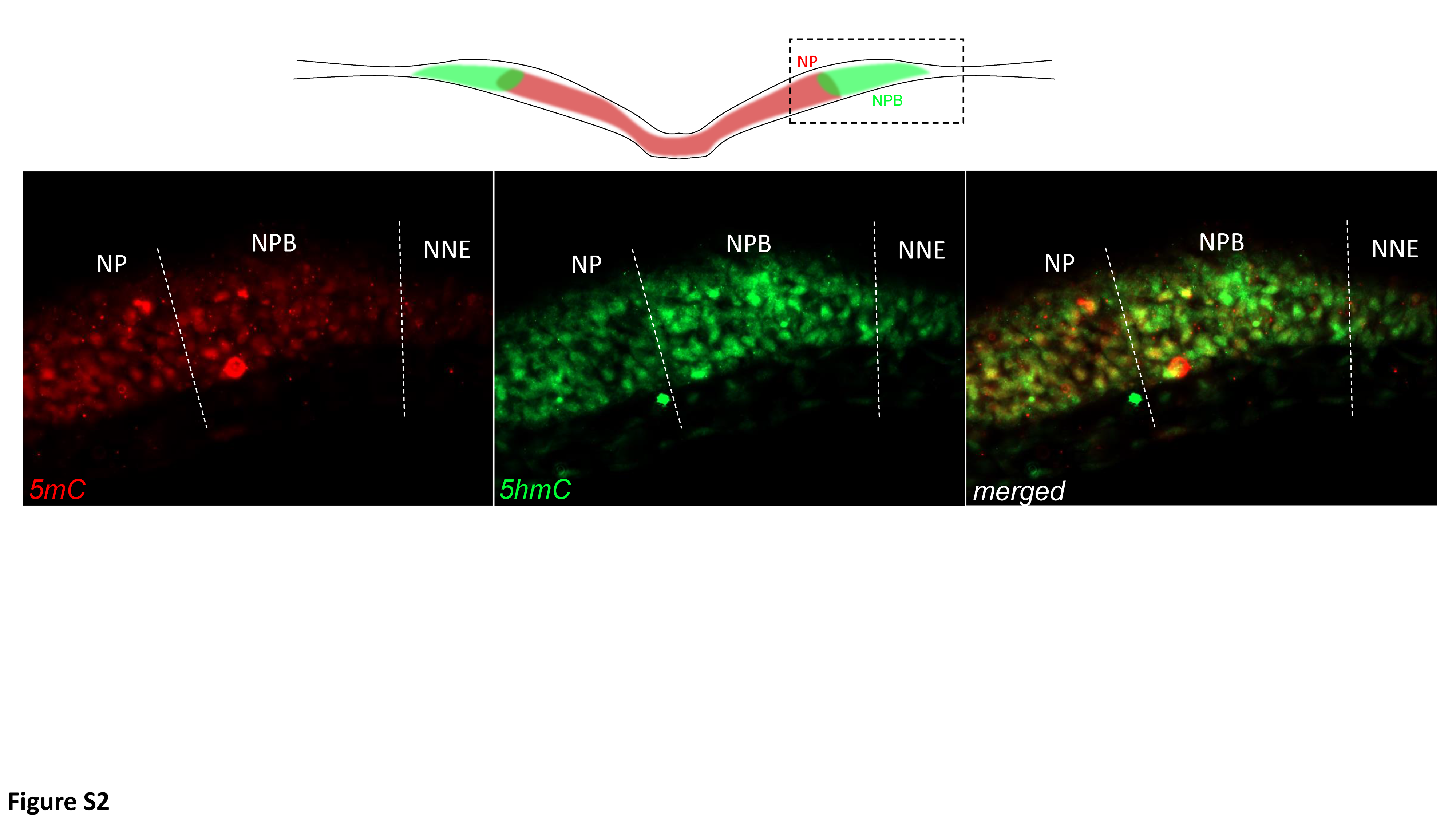

Supplement: Supplementary file 5 [file Image2.TIFF]

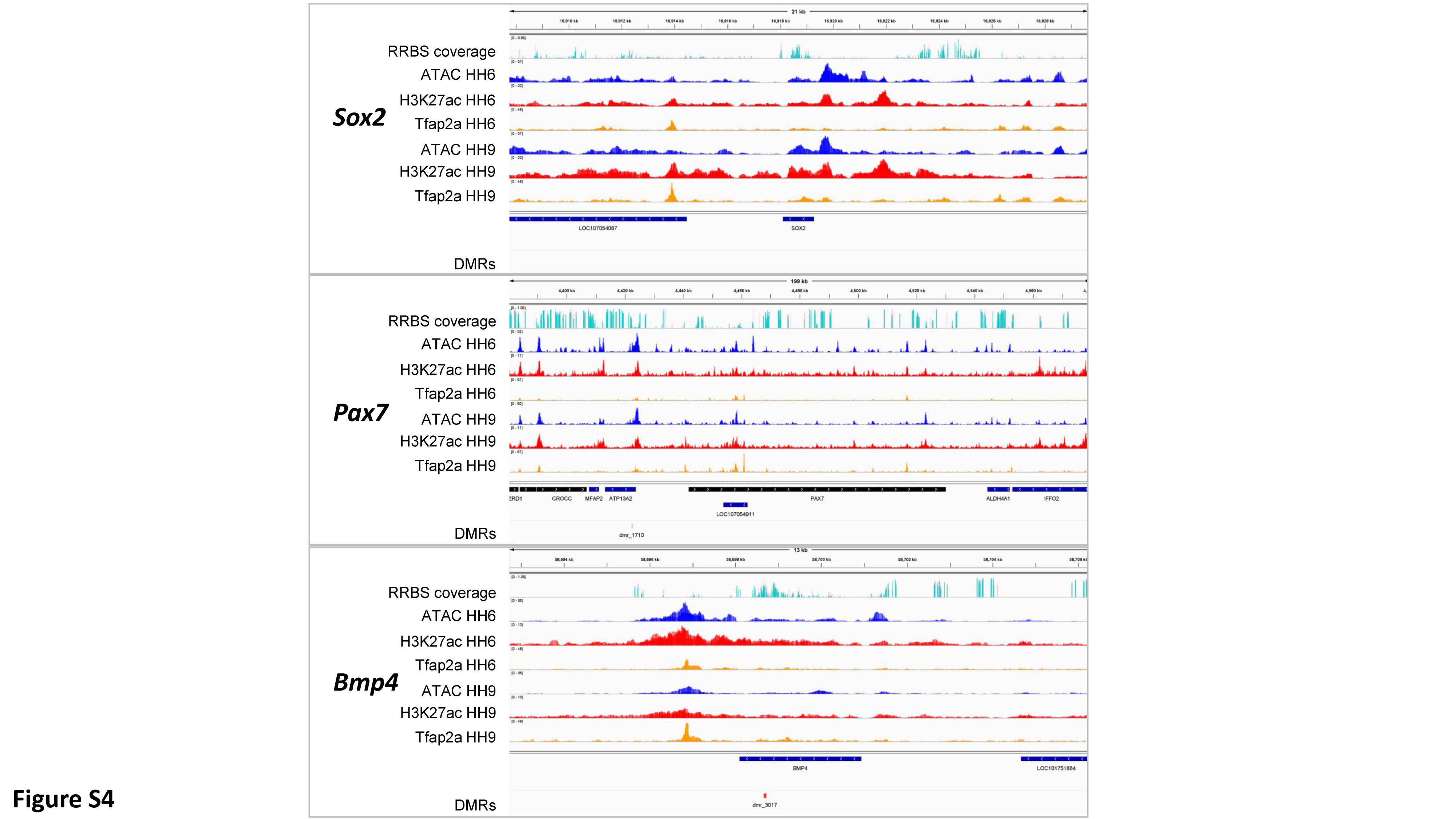

Supplement: Supplementary file 6 [file Image4.TIFF]
